# TLR3, TLR4 and TLRs7–9 Induced Interferons Are Not Impaired in Airway and Blood Cells in Well Controlled Asthma

**DOI:** 10.1371/journal.pone.0065921

**Published:** 2013-06-18

**Authors:** Annemarie Sykes, Michael R. Edwards, Jonathan Macintyre, Ajerico del Rosario, Vera Gielen, Jennifer Haas, Onn Min Kon, Mark McHale, Sebastian L. Johnston

**Affiliations:** 1 National Heart & Lung Institute, Imperial College London, London, United Kingdom; 2 MRC & Asthma UK Centre in Allergic Mechanisms of Asthma, London, United Kingdom; 3 Centre for Respiratory Infection, Imperial College London, London, United Kingdom; 4 Imperial College Healthcare NHS Trust, London, United Kingdom; 5 Respiratory and Inflammation Research Area, AstraZeneca R&D Charnwood, Loughborough, United Kingdom; University of Medicine and Dentistry of New Jersey, United States of America

## Abstract

Defective Rhinovirus induced interferon-β and interferon-λ production has been reported in bronchial epithelial cells from asthmatics but the mechanisms of defective interferon induction in asthma are unknown. Virus infection can induce interferon through Toll like Receptors (TLR)3, TLR7 and TLR8. The role of these TLRs in interferon induction in asthma is unclear. This objective of this study was to measure the type I and III interferon response to TLR in bronchial epithelial cells and peripheral blood cells from atopic asthmatics and non-atopic non-asthmatics. Bronchial epithelial cells and peripheral blood mononuclear cells from atopic asthmatic and non-atopic non-asthmatic subjects were stimulated with agonists to TLR3, TLR4 & TLRs7–9 and type I and III interferon and pro-inflammatory cytokine, interleukin(IL)-6 and IL-8, responses assessed. mRNA expression was analysed by qPCR. Interferon proteins were analysed by ELISA. Pro-inflammatory cytokines were induced by each TLR ligand in both cell types. Ligands to TLR3 and TLR7/8, but not other TLRs, induced interferon-β and interferon-λ in bronchial epithelial cells. The ligand to TLR7/8, but not those to other TLRs, induced only type I interferons in peripheral blood mononuclear cells. No difference was observed in TLR induced interferon or pro-inflammatory cytokine production between asthmatic and non-asthmatic subjects from either cell type. TLR3 and TLR7/8,, stimulation induced interferon in bronchial epithelial cells and peripheral blood mononuclear cells. Interferon induction to TLR agonists was not observed to be different in asthmatics and non-asthmatics.

## Introduction

Asthma exacerbations are an important cause of morbidity, mortality and health care costs [1]. The most common precipitant of asthma exacerbations are respiratory virus infections, particularly rhinoviruses [2], [3]. Virus infection results in induction of antiviral interferons (IFNs), including type I: IFN-α and IFN-β and type III: IFN-λ1 and IFN-λ2/3, from airway cells [4] and defective rhinovirus induced IFN production has been identified in bronchial epithelial cells (BECs) [5]–[7], bronchoalveolar lavage (BAL) cells [5], [8] and peripheral blood mononuclear cells (PBMCs) [9] from asthmatic subjects. The mechanisms causing this impaired IFN induction in cells from asthmatic subjects are unknown.

Virus induced IFN production occurs via two groups of pattern recognition receptors, Toll-like receptors (TLRs) and RNA helicases [10], [11]. Much has been learned in animal models [12], [13] but little is known about mechanisms of induction of IFNs in primary cells from humans with asthma. We have previously reported RIG-I, MDA5 and TLR3 mediate rhinovirus induction of IFNs in primary human BECs from healthy subjects [14] and that in BAL cells from asthmatics demonstrating deficient rhinovirus induced type I IFN production, TLR3, RIG-I and MDA5 expression was not different from non-asthmatics [8]. All 10 known human TLRs are present on BECs [15], however, with the exception of the known role for TLR3 in healthy subjects [14], their role in IFN induction in healthy or asthmatic subjects is unknown. TLR3, TLR7 and TLR8 have been implicated in responses to virus infection in animal models [16], TLR3 through recognition of dsRNA [12] and TLR7 and TLR8 through recognition of ssRNA and small nucleoside analogues [17].

Most work on TLR function in human asthma has been performed on PBMCs which are often used by investigators as a surrogate for airway non-structural cell function. In healthy subjects IFN-α is induced by stimulation with TLR3, TLR4, TLR7, TLR8 and TLR9 [18], [19] and IFN-β is known to be induced following TLR3 stimulation [20]. TLR7 stimulation is known to induce type III IFN in PBMCs [21] and reduced TLR7-ligand induced OAS and MxA mRNA expression and CXCL10/IP-10 protein production has been reported in PBMCs from adolescent asthmatic compared with healthy subjects [22]. However, other than TLR7, little is known about the TLRs involved in induction of type III IFNs in PBMCs and the functional role of other TLRs in PBMCs from asthmatic subjects is also uncertain. In addition to increased susceptibility to respiratory virus infection, asthmatics have increased carriage of atypical bacteria [23], [24] and increased susceptibility to invasive pneumococcal disease [25]–[27]. TLR4 and TLR9 recognise products of bacterial infection and little is known about human primary BEC IFN responses to TLR4 and TLR9 ligands in normal healthy subjects or in asthmatic subjects.

This study aimed to characterise the type I and III IFN and pro-inflammatory cytokine responses to TLR3, TLR4, TLR7, TLR8 and TLR9 stimulation in primary human BECs and PBMCs in healthy normal individuals and atopic asthmatic subjects.

## Materials and Methods

### Ethics Statement

The study was approved by the St. Mary’s Hospital Ethics Committee. Reference 07/Q0403/20.

### Methods

Primary human BECs were successfully obtained from 10 asthmatics and 9 non-asthmatic and PBMCs from 19 asthmatics and 17 non-asthmatics. Asthma and atopy was defined as previously described [8]. Smokers and subjects who had had exacerbations or respiratory tract infections in the preceding 6 weeks were excluded. Non-asthmatics had no relevant medical history, no bronchial hyperresponsiveness and were non-atopic. The study was approved by the St. Mary’s Hospital Ethics Committee. All subjects gave written informed consent.

### BEC and PBMC Isolation and Processing

BECs were obtained by fibre-optic bronchoscopy using a Keymed BF260 bronchoscope (Olympus, UK) and 5 mm sheathed endobronchial brushes (Olympus BC-202D-5010) according to BTS guidelines. Freshly obtained BECs were then seeded into bronchial epithelial growth medium (BEBM, Lonza) further supplemented with 5,000 units penicillin and 5000 µg streptomycin. Epithelial origin of cells was confirmed by cytokeratin 19 staining and cell culture was performed as previously described [7]. PBMCs were isolated from fresh whole blood and processed as previously described [4] **.**

### TLR Stimulation

TLR3 stimulation was performed with Polyinosinic-Polycytidylic Acid (Poly I:C), TLR4 with Lipopolysaccharide (LPS), TLR7/8 with Resiquimod (R848), TLR8 with RNA40 complexed with N-[1-(2,3-Dioleoyloxy)propyl]-N,N,N-trimethylammonium methyl-sulphate (DOTAP) (ssRNA) and TLR9 with CpG-ODN. For doses and source of TLR agonists see Table S1. Doses used were based on preliminary dose response experiments in commercially obtained primary bronchial epithelial cells and PBMCs (see Table S2). As negative controls, cells were treated with medium alone. Cells were harvested at 8 h, 24 h and 48 h post TLR stimulation.

### RNA Isolation and qPCR

RNA extraction was performed using RNeasy MiniKit (Qiagen, Hilden, Germany) and used for cDNA synthesis by Omniscript RT kit (Qiagen). Reactions were analysed using an ABI 7000 TaqMan (ABI Foster City, CA, USA) as previously described [14] and were normalised to 18 s. Primer and probe sequences are listed in Table S3.

### IFN Protein Measurement

IFN-β (Invitrogen, CA, USA), IFN-α (Invitrogen) and IFN-λ (R&D systems) release were measured by ELISA according to the manufacturer’s instructions. The sensitivities of each assay were 10 pg/mL.

### IL-6 and IL-8 Protein Measurement

Paired antibodies (R&D Systems) were used for IL-6 and IL-8 protein release. The sensitivities of each assay were 15 pg/mL.

### Statistical Analysis

Graphpad prism software (version 5.04; GraphPad Software, San Diego, Ca) was used to perform statistical analysis. Kruskal Wallis test with Dunn’s correction was used as the majority of data was not normally distributed. If significant, this was followed by between group testing with Mann Whitney test. A p-value of <0.05 was considered significant. Data is presented as medians unless indicated otherwise.

## Results

### IFN and Pro-inflammatory Cytokine Induction by TLR Agonists in Primary Human BECs

BECs were successfully cultured from 10 asthmatic and 9 non-asthmatic healthy subjects. Clinical characteristics of these participants are shown in Table S4. To assess which TLRs were important in IFN induction in bronchial epithelial cells, BECs were stimulated with agonists to TLR3 (poly I:C), TLR4 (LPS), TLR7/8 (R848), TLR8 (RNA40 complexed with DOTAP) and TLR9 (CpG-B-ODN and CPG-C-ODN) and supernatants and cells were harvested after 8 and 24 hrs. As the major IFN sub-types induced by virus infection of BECs are IFN-β and IFN-λ [4], investigations were limited to these sub-types. There was no statistically significant induction of either IFN-β or IFN-λ protein release or mRNA expression following TLR4, TLR8 or TLR9 stimulation at any time point (data not shown).

### TLR3 Stimulation Induced Type I and III IFN Protein Release from Human Primary BECs from Asthmatic and Healthy Subjects and TLR7/8 Stimulation Induced Type I IFN Protein in Asthmatics only and Type III IFN Protein from both Asthmatics and Non- asthmatics

**Figure 1:**
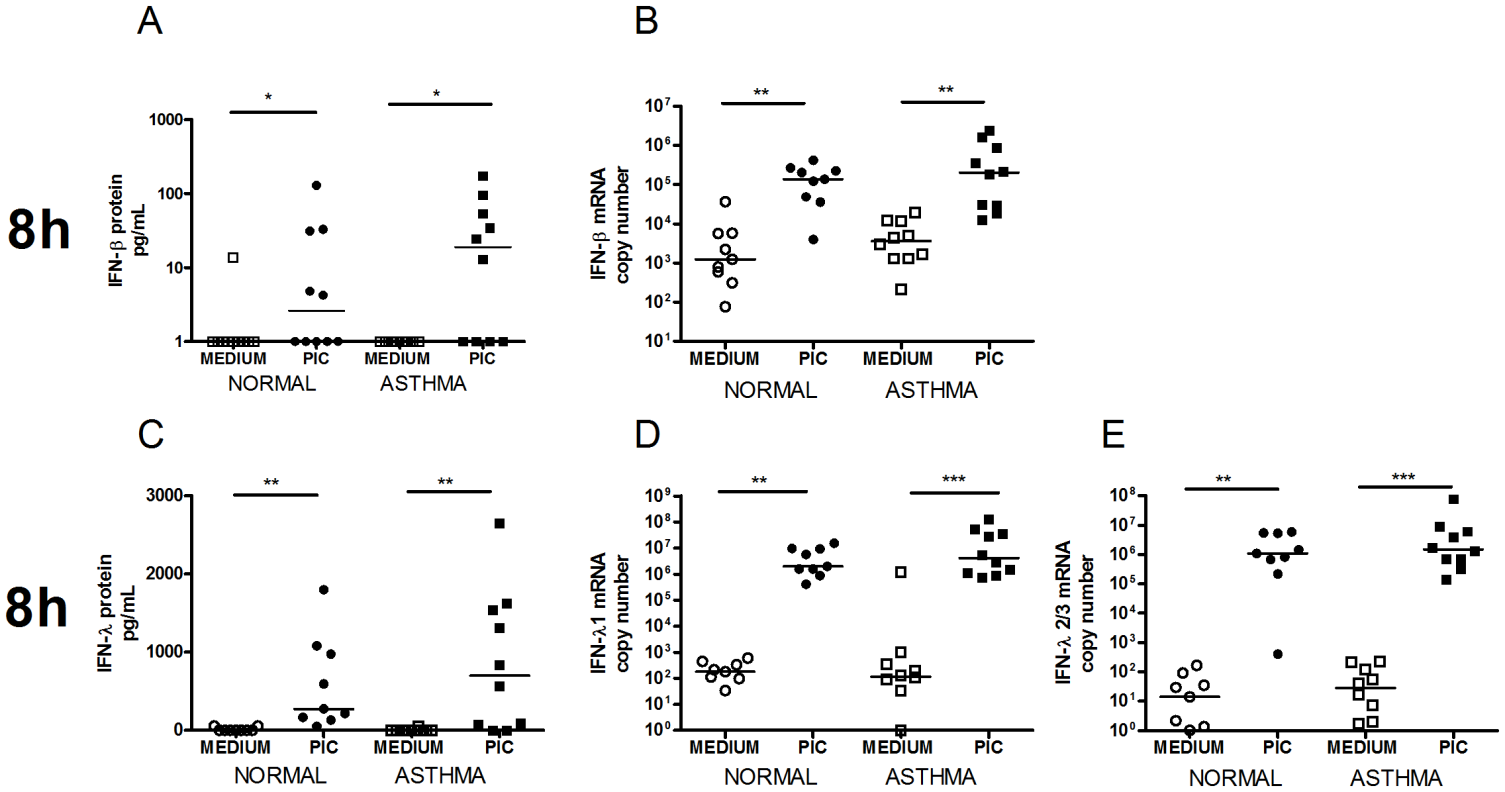
TLR3 induced type I and III IFN in HBECs. Figures depict IFN-β protein [A], IFN-β mRNA [B], IFN-λ protein [C], IFN-λ1 mRNA [D] and IFN-λ2/3 mRNA [E] at 8 h following Poly I:C stimulation. Squares represent asthmatics and circles represent non-asthmatics. * = p<0.05, ** = p<0.01, *** = p<0.001.

TLR3 stimulation induced significant type I and III IFN in BECs and induction was greatest at 8 h following stimulation. IFN-β and IFN-λ protein and mRNA were significantly induced at 8 h in both groups (Figure 1, 24 h not shown). TLR3 induced type I and III IFN protein and mRNA was not significantly different between asthmatic and non-asthmatic subjects.

**Figure 2:**
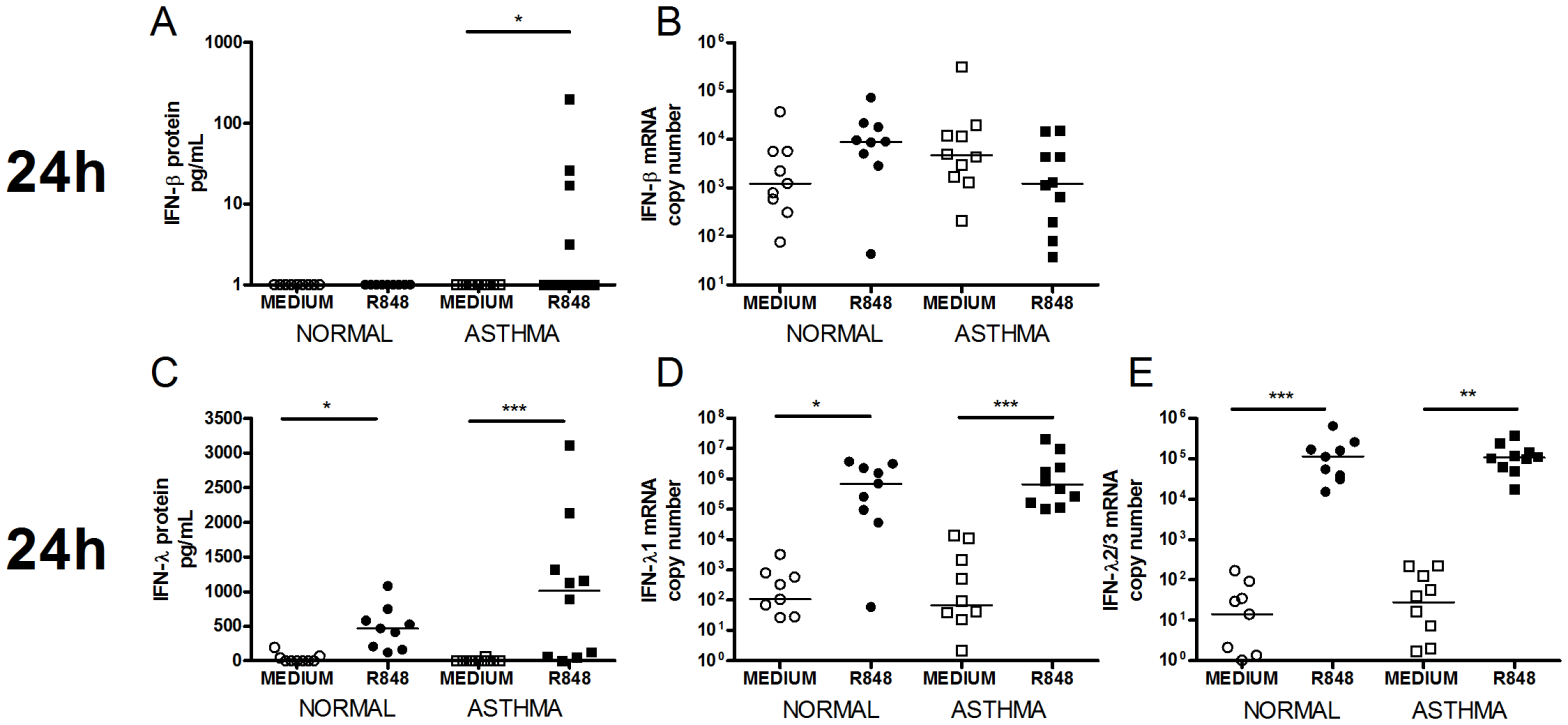
TLR7 stimulation induced type I and III IFN in HBECs. Graphs depict IFN-β protein [A], IFN-β mRNA [B], IFN-λ protein [C], IFN-λ1 mRNA [D] and IFN-λ2/3 mRNA [E] at 24 h following stimulation with R848. Squares represent asthmatics, circles represent non-asthmatics. * = p<0.05, ** = p<0.01, *** = p<0.001.

IFN response to TLR7/8 stimulation was most prominent at 24 h and consisted mainly of type III IFN. At 24 h, R848 induced some IFN-β protein in 4 asthmatic subjects only but did not induce IFN-β mRNA in either group at any time point. In the same cells at 24 h, R848 induced robust IFN-λ protein and mRNA in both groups (Figure 2, 8 h not shown). No difference was observed in IFN-λ induction between asthmatic and non-asthmatic subjects.

### Stimulation of TLR3, TLR4 and TLRs7–9 Induced Pro-inflammatory Cytokine (IL-6 and IL-8) Release from BECs in both Asthmatic and Non-asthmatic Subjects

**Figure 3:**
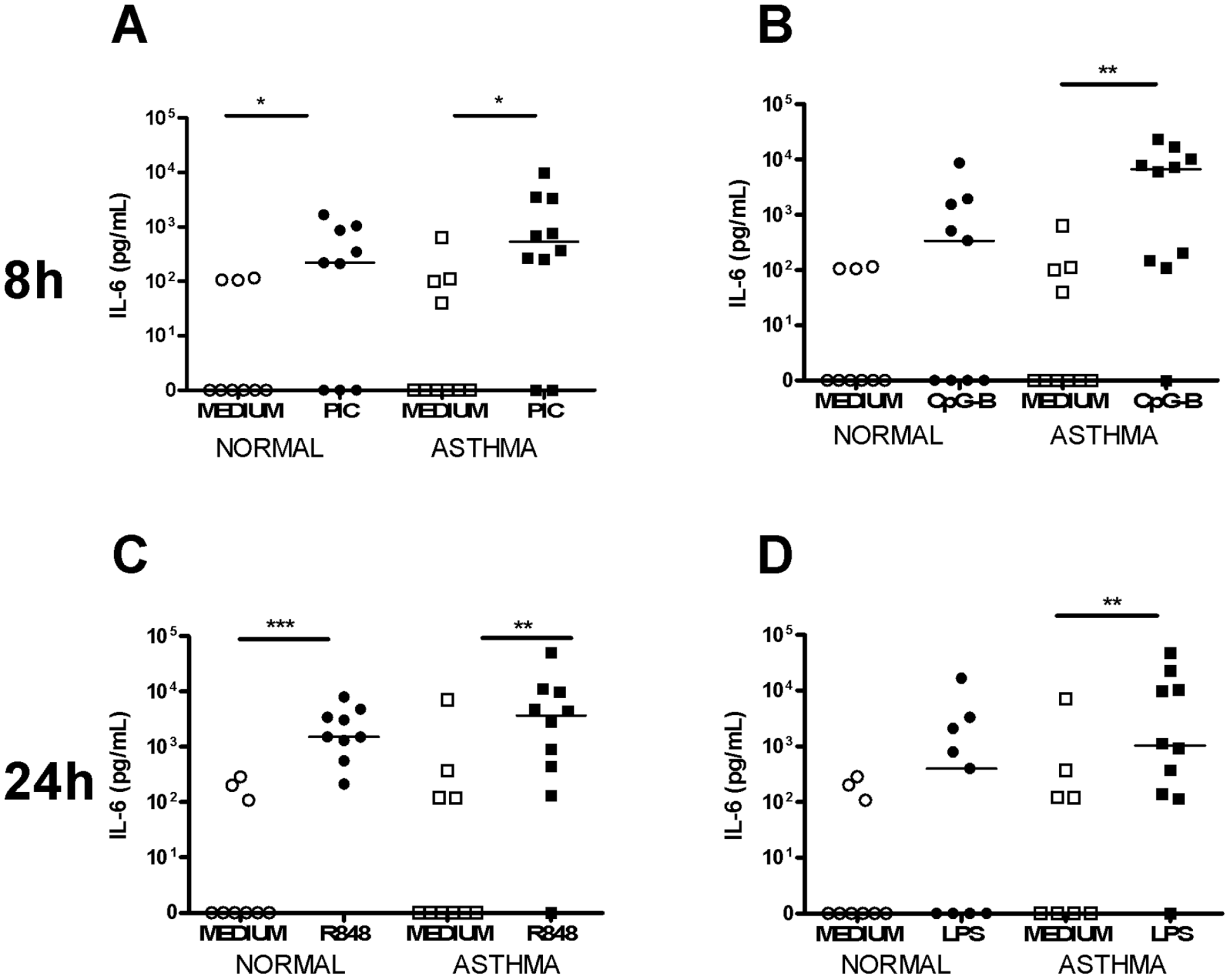
IL-6 induction following TLR stimulation in HBECs. Graphs depict IL-6 production at 8 h following stimulation with PIC [A] and CpG-B-ODN [B] and at 24 h following R848 [C] and LPS [D]. Squares represent asthmatics, circles represent non-asthmatics. * = p<0.05, ** = p<0.01, *** = p<0.001.

To confirm the TLR agonists used induced pro-inflammatory cytokines, IL-6 and IL-8 protein responses to TLR stimulation were measured. At 8 h IL-6 protein was significantly induced by poly I:C in both groups and by CpG-B-ODN in asthmatics only. At 24 h IL-6 protein was significantly induced by R848 in both groups and by LPS in asthmatics only. There was no significant IL-6 protein induction with CpG-C-ODN or RNA40 at either time point (Figure 3).

**Figure 4:**
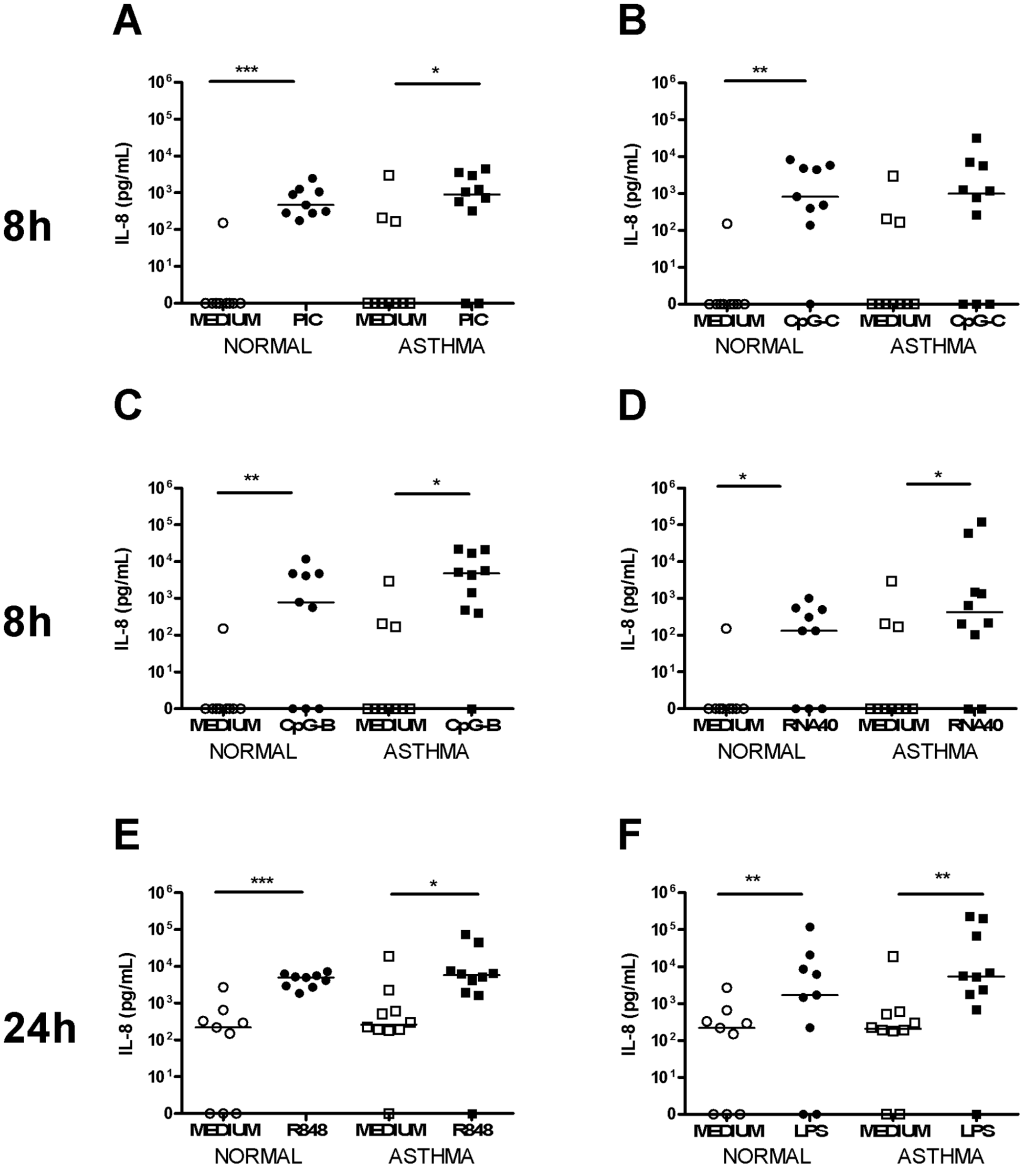
IL-8 induction following TLR stimulation in HBECs. Graphs depict IL-8 production at 8 h following stimulation with Poly I:C [A], CpG-C-ODN [B], CpG-B-ODN [C] and RNA40 [D] and at 24 h following R848 [E] and LPS [F]. Squares represent asthmatics, circles represent non-asthmatics. * = p<0.05, ** = p<0.01, *** = p<0.001.

IL-8 induction in BECs occurred following each of the TLR agonists used. Significant IL-8 protein induction was seen at 8 h with poly I:C, CpG-B-ODN and RNA40 in both groups and with CpG-C-ODN in non-asthmatics. At 24 h both LPS and R848 induced significant IL-8 protein in both groups (Figure 4). There were no significant differences between asthmatics and non-asthmatics in induction of IL-6 or IL-8 protein with any of the TLR ligands at either time point.

### IFN and Pro-inflammatory Cytokine Induction by TLR Agonists in PBMCs

To establish whether induction was similar in BECs and PBMCs, PBMCs were stimulated with the same TLR agonists detailed above and IFN protein induction at 8 h, 24 h and 48 h assessed. IFN protein induction was only observed following TLR7/8 stimulation in PBMCs, neither type I nor type III IFN protein was induced with any other TLR ligand at any time point (data not shown). As no IFN protein induction was observed with most TLR agonists, IFN mRNA expression was not assessed.

### TLR7/8 Stimulation Induced Type I but not type III IFN Induction and Stimulation of TLR3, TLR4 and TLRs 7–9 Induced Pro-inflammatory Cytokine Release from PBMCs in both Asthmatics and Non-asthmatics

**Figure 5:**
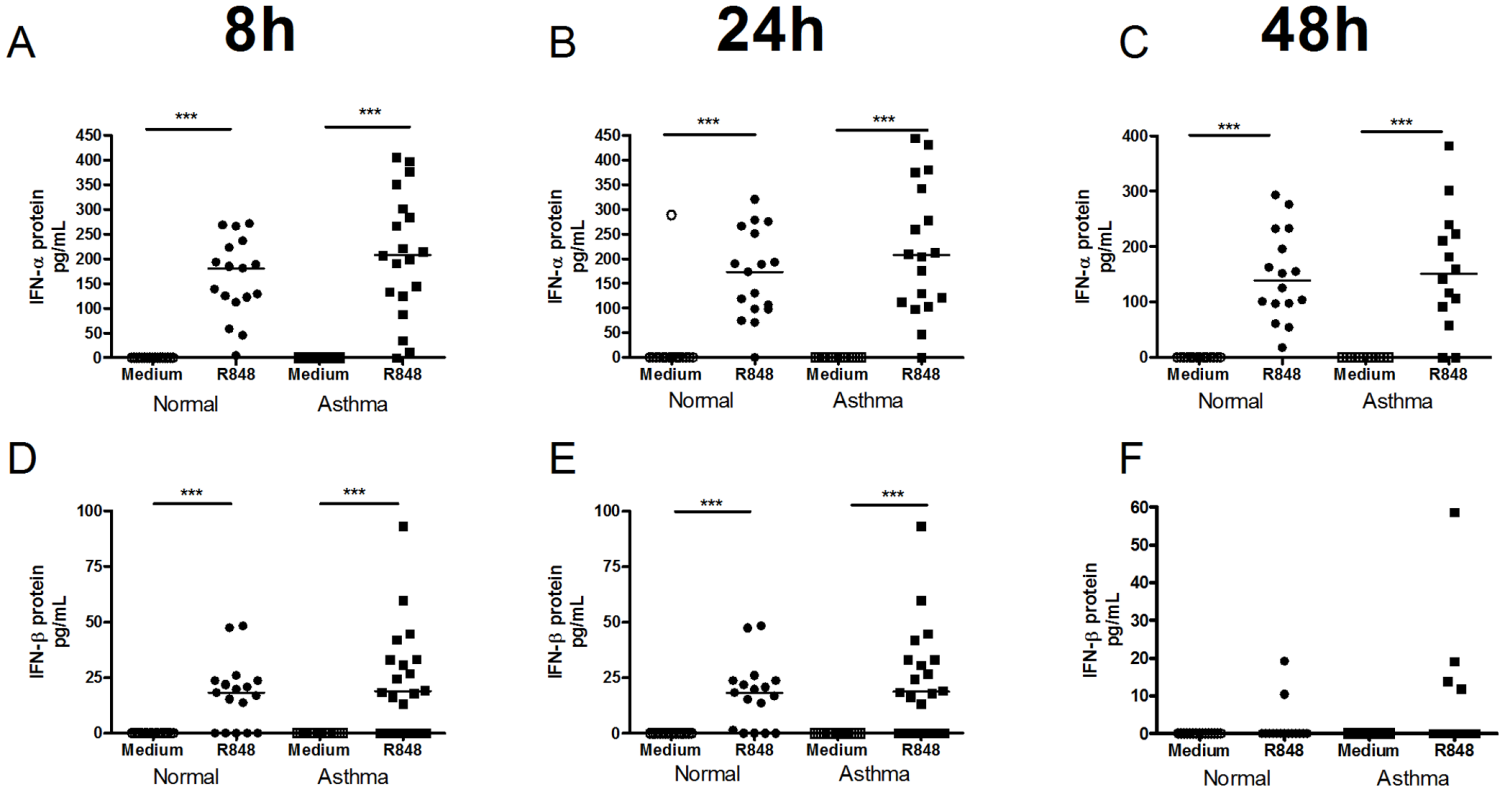
TLR7 stimulation induced type I but not type III IFN in PBMCs. Graphs depict IFN-α protein production 8 h [A], 24 h [B] and 48 h [C] and IFN-β protein at 8 h [D], 24 h [E] and 48 h [F] post R848 stimulation. Squares represent asthmatics, circles represent non-asthmatics. * = p<0.05, ** = p<0.01, *** = p<0.001.

R848 induced significant IFN-α protein release at 8 h, 24 h and 48 h in both subject groups. IFN-β protein was also induced by R848 at 8 h and 24 h but not 48 h (Figure 5). IFN-λ protein was not significantly induced at any time point (not shown). No significant difference in type I IFN induction was observed between asthmatic and normal subjects following R848 stimulation at any time point.

### Stimulation of TLR3, TLR4 and TLRs7–9 Induced Pro-inflammatory Cytokine Release from PBMCs in both Asthmatic and Non-asthmatic Subjects

**Figure 6:**
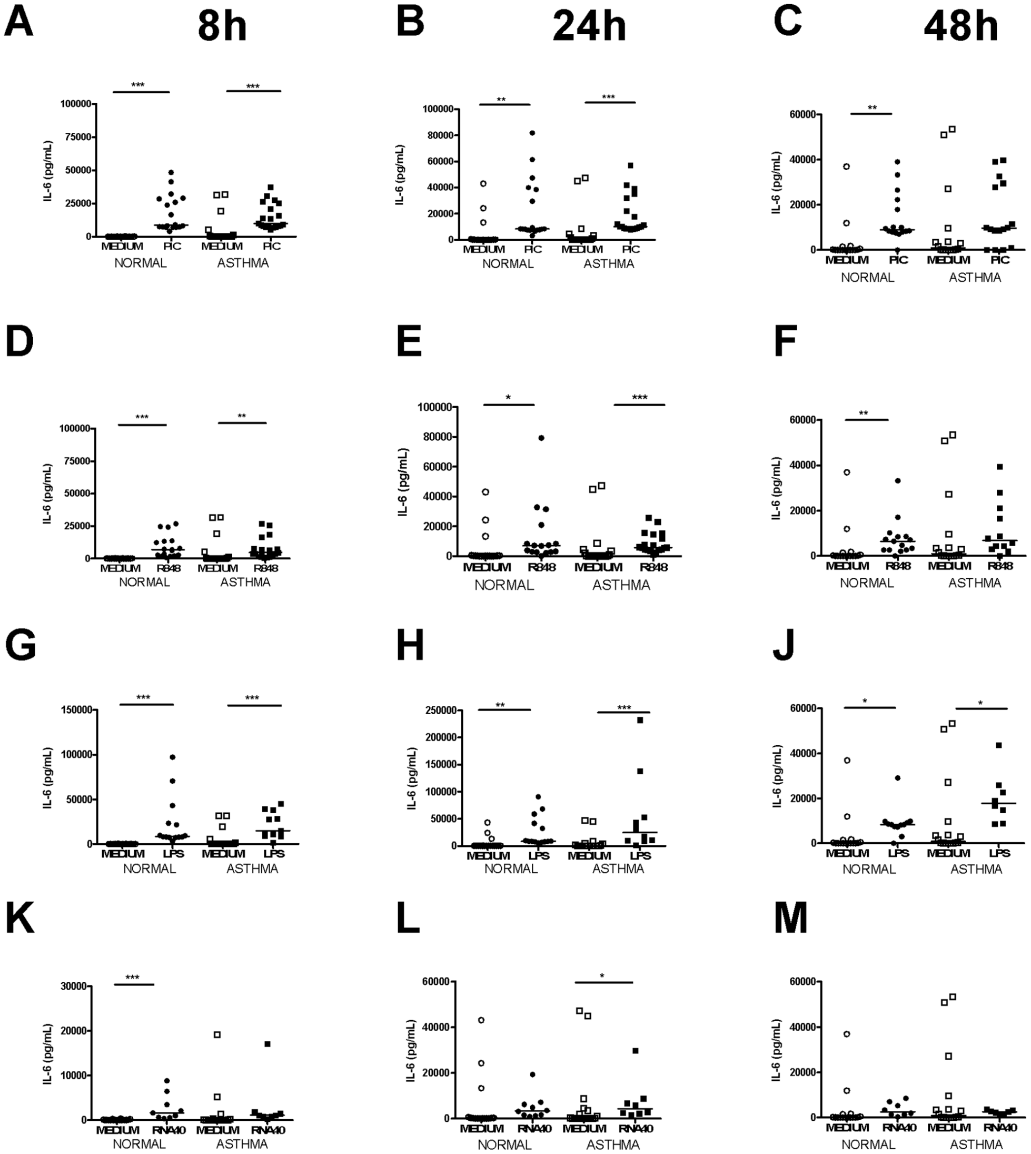
IL-6 induction following TLR stimulation in PBMCs. Graphs depict IL-6 production at 8 h following stimulation with PIC [A], R848 [D], LPS [G] and LPS [K], 24 h following PIC [B], R848 [E], LPS [H] and LPS [L] and 48 h following PIC [C], R848 [F], LPS [J] and LPS [M]. Squares represent asthmatics, circles represent non-asthmatics. * = p<0.05, ** = p<0.01, *** = p<0.001.

IL-6 protein was induced following poly I:C, LPS and R848 at 8 h, 24 h and 48 h and RNA40 at 8 h and 24 h. Significant IL-6 protein induction was observed at 8 h after stimulation with poly I:C and R848 in both groups and with LPS and RNA40 in non-asthmatics only. At 24 h significant induction was observed following stimulation with poly I:C, R848 and LPS in both groups and RNA40 in asthmatics only. At 48 h significant induction of IL-6 protein was observed following stimulation with poly I:C and R848 in non-asthmatics and LPS in both groups (Figure 6). No significant induction with either CPG occurred at any time point (not shown).

**Figure 7:**
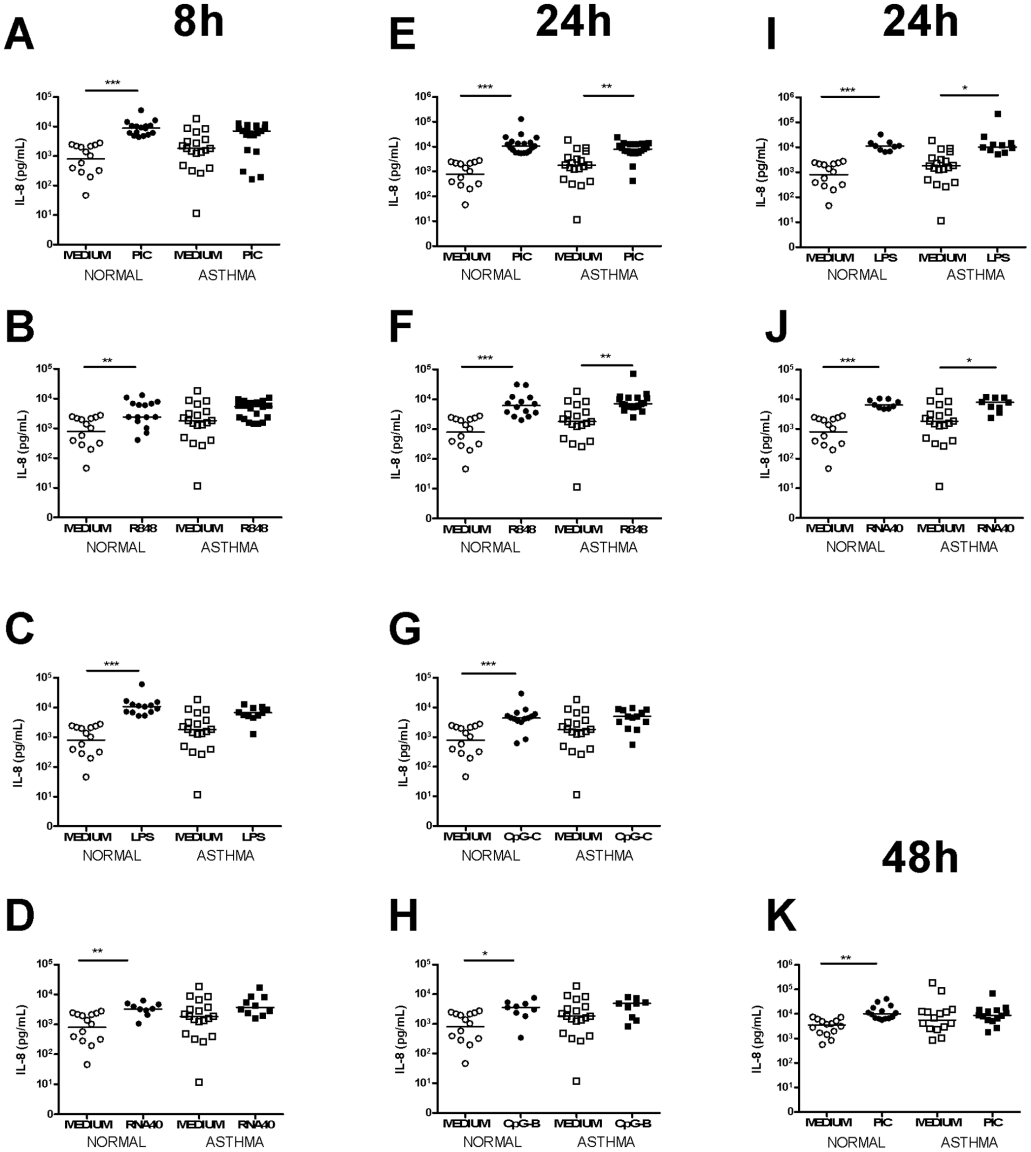
IL-8 induction following TLR stimulation in PBMCs. Graphs depict IL-8 production at 8 h following stimulation with PIC [A], R848 [B], LPS [C] and RNA40 [D], 24 h following PIC [E], R848 [F], CpG-C-ODN [G], CpG-B-ODN [H], LPS [I] and RNA40 [J] and 48 h following PIC [K]. Squares represent asthmatics, circles represent non-asthmatics. * = p<0.05, ** = p<0.01, *** = p<0.001.

IL-8 protein was induced following stimulation with all the TLR ligands. At 8 h significant IL-8 protein induction was observed in non-asthmatics following stimulation with poly I:C, R848, LPS and RNA40. At 24 h significant IL-8 protein induction was observed following stimulation with poly I:C, LPS, R848 and RNA40 in both groups and CpG-C-ODN, CpG-B-ODN in non-asthmatics only. At 48 h IL-8 protein was only significantly induced by poly I:C in non-asthmatics (Figure 7). No significant induction was seen with these ligands at other time points (not shown) and there was no significant difference in IL-6 or IL-8 protein induction between non-asthmatic and asthmatic subjects with any ligand at any time point.

## Discussion

Viruses are responsible for the majority of asthma exacerbations and can induce type I and III IFNs via TLRs. Type I and III IFN induction is deficient in asthma. TLR agonists have been identified as potential therapeutic options for asthma but, despite much work in animal models, little was previously known about IFN responses of airway cells to TLR agonists in human asthma. TLR3 and TLR7/8, were identified as the predominant IFN inducers in BECs, inducing both type I and III IFN. TLR7/8 was identified as the predominant inducer of IFN in PBMCs inducing only type I IFN, a different pattern to that observed in BECs. No difference was observed in TLR induced IFN or pro-inflammatory cytokine production between asthmatic and non-asthmatic subjects from either BECs or PBMCs.

In these experiments ligation of TLR7/8, but not TLR8 alone, induced robust type III IFN but minimal type I IFN in BECs. R848 is known to stimulate both TLR7 and TLR8 with TLR7 showing a higher sensitivity to R848 than TLR8 [20] As no induction was observed following TLR8 stimulation it is possible that the robust type III IFN response observed is due to stimulation of TLR7. Although statistically significant IFN-β protein induction occurred in asthmatic subjects, there was no mRNA induction and it was only identified in 4 subjects. This increased responsiveness of R848 in the asthmatic cells is also surprising in the context of previous reports of reduced TLR7 function in PBMCs in asthma [22] and in pregnancy [21] and should be interpreted with caution. Induction of IFN-λ protein and mRNA was clearly robust suggesting that R848 may be more important in the production of IFN-λ than IFN-β in BECs. It is currently unclear why R848 stimulation should predominantly induce type III IFN, over type I IFN, in this cell type.

Due to the invasive nature of obtaining bronchial epithelial cells PBMCs are often used as a surrogate for airway cells. R848 stimulation in PBMCs produced robust type I IFN induction but no IFN-λ, the opposite pattern to that observed in BECs and suggests that TLR7 predominantly produces type I IFN in PBMCs. In addition to different patterns of IFN subtype induction, timing of induction was also different. The differences in IFN induction in PBMCs and BECs from the same individuals in response to both TLR3 and TLR7/8 agonists highlight that using PBMCs as a surrogate for BECs may be sub-optimal and misleading with regard to responses in BECs.

IFN-α induction by PBMCs from healthy individuals has previously been reported following stimulation with TLR3, TLR4, TLR7, TLR8 and TLR9 [18], [19] and IFN-β following TLR3 stimulation [20]. Differences exist in the experimental techniques used in these studies, including the use of pooled, stored PBMCs [19] and different agonists [18], doses [20] and cell numbers [20] which may influence the differences in observed findings. Previous studies also used samples from smaller numbers of individuals and these discrepant findings further highlight the problems of using PBMCs to represent airway cell function.

There have now been multiple reports of defective virus induced IFN induction in various cell types from people with asthma [5]–[9], [28]–[31]. As TLRs induce IFN in response to virus infection it was possible that asthmatics may have had defective TLR function which may result in deficient IFN induction. The experiments performed identified that TLR function was similar in these asthmatic and non-asthmatic subjects, except for the weak but statistically significant induction of IFN-β protein that was observed in BECs from asthmatic but not normal subjects. Despite this, it can be concluded that in the BECs and PBMCs in this study asthmatic subjects did not have deficient TLR induced IFN responses when compared to normal subjects. These experiments were planned at a time when defective IFN induction in asthma was limited to 4 reports [5], [7], [28], [29], none of which reported relationships with asthma severity or control. Since recruitment completed, some evidence has emerged suggesting that IFN-deficiency may be more profound in more severe active asthma [8], [30]. The asthmatics recruited in this study were well controlled, median ACQ was 0.59 with good asthma control being defined as an ACQ score of <0.75 [32], which may have influenced the failure to detect defective IFN induction.

In summary, this study investigated the role of TLRs 3, 4 and 7–9 in type I and type III IFN production in BECs and PBMCs from asthmatic and healthy non-asthmatic subjects. Stimulation of TLR3 and TLR7/8, but not TLR4 or 9, induced IFNs in BECs. No differences were detected in IFN induction between asthmatic and non-asthmatic subjects following TLR stimulation.

## Supporting Information

Table S1 TLR agonists and doses used for HBECs and PBMCs.
(DOCX)

Table S2 Doses of TLR agonists used in preliminary experiements.
(DOCX)

Table S3 Primer and probe sequences used.
(DOCX)

Table S4 Clinical characteristics of participants from whom HBEC samples were successfully obtained.
(DOCX)

Methods S1 **Preliminary TLR agonist dosing experiments.**
(DOCX)
