# Supplementary material for: TLR3, TLR4 and TLRs7–9 Induced Interferons Are Not Impaired in Airway and Blood Cells in Well Controlled Asthma
Source: PLoS One. 2013 Jun 18;8(6):e65921. doi: 10.1371/journal.pone.0065921 (PMC3688823; doi:10.1371/journal.pone.0065921)
Supplement: Table S1 — TLR agonists and doses used for HBECs and PBMCs. (DOCX) [file pone.0065921.s001.docx]

| Ligand | Receptor | Source | Doses  HBECs PBMCs | |
| --- | --- | --- | --- | --- |
| Polyinosinic-Polycytidylic Acid (Poly IC) | TLR3 | Sigma Aldrich | 10 mg/mL | 100 mg/mL |
| Lipopolysaccharide (LPS) | TLR4 | Sigma Aldrich | 10 mg/mL | 10 mg/mL |
| Resiquimod (R848) | TLR7 | Eurogentec | 1μM | 1μM |
| RNA40 (ssRNA) | TLR8 | Eurogentec | 50 μg/mL | 50 μg/mL |
| CpG-B-ODN | TLR9 | Eurogentec | 3μM | 3μM |
| CpG-C-ODN | TLR9 | Eurogentec | 3μM | 3μM |

**Table S1.** TLR agonists and doses used for HBECs and PBMCs
