# Supplementary material for: TLR3, TLR4 and TLRs7–9 Induced Interferons Are Not Impaired in Airway and Blood Cells in Well Controlled Asthma
Source: PLoS One. 2013 Jun 18;8(6):e65921. doi: 10.1371/journal.pone.0065921 (PMC3688823; doi:10.1371/journal.pone.0065921)
Supplement: Table S2 — Doses of TLR agonists used in preliminary experiements. (DOCX) [file pone.0065921.s002.docx]

| TLR Agonist | Doses used |
| --- | --- |
| Poly IC | 0.1, 1, 10, 50, 100 & 200 mg/mL |
| R848 | 0.1, 1 & 10 μM |
| CpG-B-ODN | 0.1, 0.3, 1, 3, 10 & 30 μM |
| CpG-C-ODN | 0.1, 0.3, 1, 3, 10 & 30 μM |
| LPS | 1, 10 & 100 mg/mL |
| RNA40 | 1, 10, 50 & 100 μg/mL |
| RNA40 + DOTAP | 1, 10, 50 & 100 μg/mL |
