# Supplementary material for: TLR3, TLR4 and TLRs7–9 Induced Interferons Are Not Impaired in Airway and Blood Cells in Well Controlled Asthma
Source: PLoS One. 2013 Jun 18;8(6):e65921. doi: 10.1371/journal.pone.0065921 (PMC3688823; doi:10.1371/journal.pone.0065921)
Supplement: Table S3 — Primer and probe sequences used. (DOCX) [file pone.0065921.s003.docx]

| 18S Forward (300nM) | CGCCGCTAGAGGTGAAATTCT |
| --- | --- |
| 18S Reverse (300nM) | CATTCTTGGCAAATGTCG |
| 18S Probe | ACCGGCGCAAGACGGACCAGA |
| IFN-α(A) Forward (300nM) | CAGAGTCACCCATCTCAGCA |
| IFN-α(A) Reverse (900nM) | CACCACCAGGACCATCAGTA |
| IFN-α(A) Probe | ATCTGCAATATCTACGATGGCCTCGCC |
| IFN-α(B) Forward (300nM) | CTGGCACAAATGGGAAGAAT |
| IFN-α(B)Reverse (900nM) | CTTGAGCCTTCTGGAACTGG |
| IFN-α(B) Probe | TTTCTCCTGCCTGAAGGACAGACATGA |
| IFN-β Forward (300nM) | CGCCGCATTGACCATCTA |
| IFN-β Reverse (900nM) | GACATTAGCCAGGAGGTTCTCA |
| IFN-β Probe | TCAGACAACATTCATCTAGCACTGGCTGGA |
| IFN-λ1 Forward(300nM) | GGACGCCTTGGAAGAGTCACT |
| IFN-λ1 Reverse (900nM) | AGAAGCCTCAGGTCCCAATTC |
| IFN-λ1 Probe | AGTTGCAGCTCTCCTGTCTTCCCCG |
| IFN-λ2/3 Forward (300nM) | CTGCCACATAGCCCAGTTCA |
| IFN-λ2/3 Reverse (900nM) | AGAAGCGCATCTTCTAAGGCATCTT |
| IFN-λ2/3 Probe | TCTCCACAGGAGCTGCAGGCCTTTA |
| TLR3 Forward (900nM) | AAATTAAAGAGTTTTCTCCAGGGTGTT |
| TLR3 Reverse (300nM) | ATTCCGAATGCTTGTGTTTGC |
| TLR 3 Probe | TTTGGCCTCTTTCTGAACAATGTCCAGA |
| TLR 7 Forward (300nM) | CCCTTTCAGAAGTCCAAGTCCCT |
| TLR 7 Reverse (900nM) | GGTGAGCTTGCGGGTTTGT |
| TLR 7 Probe | CTCCGGAAAAGGCTCTGTGGGAGTTC |
| TLR 8 Forward (900nM) | AATTGCTGGCCCACAGTTTT |
| TLR 8 Reverse (900nM) | CCTGAGCAGAGGACCCAGTTA |
| TLR 8 Probe | GCTGGGCCTGGACTCCTGACCCT |
