# Supplementary material for: TLR3, TLR4 and TLRs7–9 Induced Interferons Are Not Impaired in Airway and Blood Cells in Well Controlled Asthma
Source: PLoS One. 2013 Jun 18;8(6):e65921. doi: 10.1371/journal.pone.0065921 (PMC3688823; doi:10.1371/journal.pone.0065921)
Supplement: Table S4 — Clinical characteristics of participants from whom HBEC samples were successfully obtained. (DOCX) [file pone.0065921.s004.docx]

| Clinical characteristic | Asthma | Non-asthmatic | p value |
| --- | --- | --- | --- |
| Sex | 80% M 20% F | 42% M 58% F |  |
| Age | 34.2 (± 2.678) | 38.58 (± 3.209) | 0.307 |
| ACQ | 0.59 (± 0.139) | 0 (± 0) | 0.003 |
| ICS | 0.5 (± 0.166) | 0 (± 0) | 0.003 |
| ISC and LABA | 0.3 (± 0.152) | 0 (± 0) | 0.004 |
| Exacerbations / year | 1.1 (± 0.1) | 0 (± 0) | 0.004 |
| Oral steroids / year | 0.1 (± 0.1) | 0 (± 0) | 1.000 |
| PEF (L/min) | 525 (± 35.69) | 470 (± 27.06) | 0.575 |
| PEF % predicted | 95.1 (± 4.20) | 102.7 (± 3.11) | 0.086 |
| FEV_1_ (L) | 3.61 (± 0.296) | 3.38 (± 0.175) | 0.373 |
| FEV_1_% predicted | 90.4 (± 5.102) | 96.25 (± 2.77) | 0.499 |
| FVC (L) | 4.66 (± 0.349) | 3.95 (± 0.201) | 0.080 |
| FVC % predicted | 97.10 (± 4.66) | 95.50 (± 3.79) | 0.488 |
| FEV_1_/FVC ratio | 77.26 (± 1.99) | 85.77 (± 1.95) | 0.008 |
| PC_20_  (mg/mL) | 1.42 (± 0.484) | 16 (± 0) | 0.003 |
| IgE (units/mL) | 154.6 (± 37.97) | 15.1 (± 3.53) | <0.001 |
| SPT (number) | 4.1 (± 0.48) | 0 (± 0) | <0.001 |
