# Supplementary material for: TLR3, TLR4 and TLRs7–9 Induced Interferons Are Not Impaired in Airway and Blood Cells in Well Controlled Asthma
Source: PLoS One. 2013 Jun 18;8(6):e65921. doi: 10.1371/journal.pone.0065921 (PMC3688823; doi:10.1371/journal.pone.0065921)
Supplement: Methods S1 — Preliminary TLR agonist dosing experiments. (DOCX) [file pone.0065921.s005.docx]

Doses used were based on experiments with the above TLR agonists in a human bronchial epithelial cell line. Doses used were based on experiments with the above TLR agonists in primary human bronchial epithelial cells and PBMCs. Briefly, comercially obtained primary bronchial epithelial cells were cultured to a confluent monolayer and then stimulated with TLR agonists at doses listed in supplementary table 2. The doses chosen for use in the main study were based on strongest IFN and proinflammatory cytokine responses in these dose response experiemtns. RNA40 was complexed to DOTAP to enable transfection into the cell.
